# Functional Implications of the *CLOCK* 3111T/C Single-Nucleotide Polymorphism

**DOI:** 10.3389/fpsyt.2016.00067

**Published:** 2016-04-21

**Authors:** Angela R. Ozburn, Kush Purohit, Puja K. Parekh, Gabrielle N. Kaplan, Edgardo Falcon, Shibani Mukherjee, Hannah M. Cates, Colleen A. McClung

**Affiliations:** ^1^Department of Psychiatry and Translational Neuroscience Program, University of Pittsburgh School of Medicine, Pittsburgh, PA, USA; ^2^Department of Behavioral Neuroscience, Oregon Health & Science University, Portland, OR, USA; ^3^Portland Alcohol Research Center, VA Medical Center, Portland, OR, USA; ^4^Department of Pharmacology, University of Pennsylvania, Philadelphia, PA, USA; ^5^Department of Psychiatry, University of Texas Southwestern Medical Center, Dallas, TX, USA; ^6^Fishberg Department of Neuroscience, Icahn School of Medicine at Mount Sinai, New York, NY, USA

**Keywords:** circadian, clock, single-nucleotide polymorphism, gene expression, bipolar disorder, cell culture

## Abstract

Circadian rhythm disruptions are prominently associated with bipolar disorder (BD). Circadian rhythms are regulated by the molecular clock, a family of proteins that function together in a transcriptional–translational feedback loop. The CLOCK protein is a key transcription factor of this feedback loop, and previous studies have found that manipulations of the *Clock* gene are sufficient to produce manic-like behavior in mice (1). The *CLOCK* 3111T/C single-nucleotide polymorphism (SNP; rs1801260) is a genetic variation of the human *CLOCK* gene that is significantly associated with increased frequency of manic episodes in BD patients (2). The 3111T/C SNP is located in the 3′-untranslated region of the *CLOCK* gene. In this study, we sought to examine the functional implications of the human *CLOCK* 3111T/C SNP by transfecting a mammalian cell line (mouse embryonic fibroblasts isolated from *Clock^−/−^* knockout mice) with pcDNA plasmids containing the human *CLOCK* gene with either the T or C SNP at position 3111. We then measured circadian gene expression over a 24-h time period. We found that the *CLOCK*3111C SNP resulted in higher mRNA levels than the *CLOCK* 3111T SNP. Furthermore, we found that *Per2*, a transcriptional target of CLOCK, was also more highly expressed with *CLOCK* 3111C expression, indicating that the 3′-UTR SNP affects the expression, function, and stability of *CLOCK* mRNA.

## Introduction

Bipolar disorder (BD) is a severe and chronic psychiatric disease that afflicts approximately 1–3% of the United States population (3). BD incurs substantial societal burdens, including a devastating global cost of illness primarily due to patients’ lower level of functioning, greater severity of disability, longer duration of illness, and ultimately greater loss in productivity when compared patients with other mood disorders (4, 5). BD therapies currently in use include mood stabilizers, such as lithium and valproate; however, these are effective for only a portion of patients (6). The underlying cause of BD is unknown, though there is a growing body of evidence linking disruptions in circadian rhythms with the disease (7).

Disruption of sleep and circadian rhythms are common to many psychiatric disorders, including BD, which presents with severe circadian rhythm disruptions as a prominent symptom. It has been shown that in individuals with BD, mood episodes are affected by light and also follow seasonal patterns (6, 8–10). In addition to fluctuations in mood, BD patients typically exhibit irregularities in important physiological processes that are largely regulated by the body’s circadian rhythms (such as sleep, diurnal activity, body temperature, and blood pressure cycles). Mood stabilizers, such as lithium, are known to restore some of these disrupted rhythms in BD patients by producing a strong phase-delay in rhythms and increasing rhythmic amplitude, which may be very important for their observed therapeutic effects (11–17).

Circadian rhythms are regulated by a molecular clock, which consists of several core “clock” genes (e.g., *Clock*, *Npas2, Bmal1*, *Per1*, and *Per2*) that are expressed throughout the body. These elements interact with each other through a series of transcriptional and translational feedback loops that are regulated over a 24-h period in the absence of environmental input (18). Within the suprachiasmatic nucleus (SCN) and other regions, circadian rhythms are controlled by the molecular clockwork, which comprises a series of autoregulatory transcriptional–translational feedback loops. The transcription factors CLOCK or NPAS2 heterodimerize with BMAL1 and regulate the transcription of target genes, including the *Period* (*Per*) and *Cryptochrome* (*Cry*) genes, which act to inhibit the activity of the CLOCK/BMAL1 complex. Several pre-clinical studies have identified an important role for circadian genes in mood-related behaviors. Recent human genetic studies have linked elements of the molecular clock to BD. Polymorphisms in *CLOCK* and other circadian genes have been found to be associated with various aspects of BD (19–25). In addition, rhythm disruptions and sleep disturbances often precipitate manic or depressive episodes (26). By studying molecular mechanisms that may underlie such BD symptoms, we can gain a better understanding of its causes and how to most effectively treat patients with this crippling disease.

The importance of circadian genes in BD has been suggested by several human genetic studies that have identified significant associations between mutations or polymorphisms of circadian genes and BD. The *Per* genes, which act as key repressors of the circadian transcriptional–translational feedback loop in humans, have been found to associate with mood disorders and their age of onset (27, 28). Recent genome-wide association studies (GWAS) have also implicated circadian modulators, such as *ARNTL* and *DEC1*, as significantly associated with BD (29). These results are particularly important given that data from family and twin studies that have established BD’s genetic heritability as up to 85%, suggesting a strong genetic component (30). Here, we investigate the role of a single-nucleotide polymorphism (SNP) in one circadian gene, *CLOCK*, that has been associated with BD (2). Interestingly, the *CLOCK* 3111T/C SNP is a genetic variant of the human *CLOCK* gene that has been associated with bipolar mania in BD patients. Specifically, this T→C polymorphism (rs1801260) associates with increased actimetric and sleep disturbances and incidences of manic episodes in bipolar patients (2, 31, 32). The prevalence of the 3111C has been reported to be between 19 and 21% in American populations (33). BD patients carrying the 3111C allele experience higher rates of insomnia and sleep disturbances, and differences between the 3111C and 3111T allele carriers were attenuated with ongoing chronic lithium treatment (32). No research group, however, has yet sought to determine the molecular mechanism through which this SNP can affect individuals with BD. The 3111T/C SNP is located within the *CLOCK* gene’s 3′-untranslated region (3′-UTR). The 3′-UTR is a region that has been shown to be very important for mRNA stability and modifications to this region can lead to either increased stability or degradation of gene products (34, 35).

Moreover, preclinical studies have shown that manipulations of the circadian gene, *Clock*, are sufficient to produce a behavioral phenotype sharing several features with bipolar mania. Mice with a dominant-negative *Clock* gene mutation exhibit a manic-like phenotype, including hyperactivity, reduced anxiety and depression-like behaviors, and increased drug and alcohol intake (1, 36–38). Similar to human BD patients, these mutant mice are responsive to chronic lithium, as well as valproate treatment (39, 40).

Together, these results have led us to hypothesize that the 3111C SNP in the *CLOCK* gene decreases *Clock* mRNA and/or protein levels, and that this could be a possible mechanism through which this polymorphism can affect mood in individuals with BD. In this study, we investigated the effects of the human 3111T/C SNP on *CLOCK* mRNA in a mammalian cell line by examining the differences between the 3111T and 3111C variants of *CLOCK*.

## Materials and Methods

### Plasmid Construction

The pBluescript II plasmid containing the full human *CLOCK* gene (product i.d. ORK00509, Kazusa), was digested with Apa1 and Not1 restriction enzymes. The DNA fragment containing the *CLOCK* gene was isolated using agarose gel electrophoresis and the QIAQuick gel purification kit (catalog no. 28704, QIAGEN). The 5kb *CLOCK* containing fragment was ligated into the pcDNA3.1(−) plasmid (catalog no. V795-20, Invitrogen) digested with *Apa1* and *Not1* restriction enzyme sites (5′ end and 3′ end, respectively) and treated with calf intestinal phosphatase to prevent self-ligation. The ligated plasmids were transformed into One-Shot TOP10 Chemically Competent *Escherichia coli* cells (catalog no. C4040-10, Invitrogen). We performed site-directed mutagenesis of the *CLOCK* construct at the site of the 3111T/C SNP using the Quikchange II XL Site-Directed Kit (catalog no. 200521, Agilent) to perform a C to T transformation, creating a 3111T version of the *CLOCK* gene 3′-UTR (forward primer: 5′-GAGGTGATCATAGGGGCATAGCCAG TTCTGACAGTG-3′, reverse primer: 5′-CACTGTCA GAACTGGCTATGCCCCTATGATCACCTC-3′). The full construct was verified through a series of restriction enzyme digests and complete sequencing of the *CLOCK* gene (including cloning junctions) by the University of Pittsburgh Genomics and Proteomics Core Laboratories. Isolated and sequence-verified clones were grown in *E. coli* and plasmids were isolated using QIAGEN’s QIAprep Spin Miniprep kit (catalog no. 27104) and endonuclease-free Plasmid Maxi Kit (catalog no. 12162).

### shRNA Construction

A small hairpin RNA (shRNA) was constructed against the *Npas2* gene by selecting a 24 base sequence (5′-GAACACTGGATTCTTCCTGTTAAC-3′) in the 3′-UTR (41). For the scrambled (Scr) shRNA, a random sequence of 24 bases (5′-CGGAATTTAGTTACGGGGATCCAC-3′) that had no sequence similarities with any known genes/mRNA was used (42). An anti-sense sequence of the selected mRNA region followed by a miR23 loop of 10 nucleotides (CTTCCTGTCA) was added at the 5′ end of the above sequences. The miR23 loop facilitates the transfer of the hairpin RNA out of the nucleus. These shRNAs were designed as synthetic duplexes with overhang ends identical to those created by *Sap*I and *Xba*I restriction enzyme digestion. The annealed oligonucleotides were cloned into the adeno-associated virus (AAV) plasmid expressing enhanced green fluorescent protein (GFP) (catalog no. 240075, Agilent Technologies).

### Preparation of Mouse Embryonic Fibroblasts

Mouse embryonic fibroblasts (MEFs) were isolated and prepared from homozygous *Clock* knockout (KO) mice at 13–14 day post-coitum (Jackson Labs, Bar Harbor, ME, USA, stock#010925) (43). All experiments were approved by the Institutional Animal Care and Use Committee and adhered to NIH Guidelines. *Clock* KO MEFs were used to avoid confounds of endogenous *Clock* gene expression when measuring expression levels in MEFs transfected with the 3111T/C constructs. Briefly, uterine horns were dissected, and embryos were individually separated. The head and red organs were removed before the embryos were finely minced using a sterile razor blade. Minced tissue was treated with 0.05% Trypsin-EDTA (Gibco, Invitrogen) and DNase I (USB), and incubated for 15 min at 37°C. Cells were centrifuged at 600 × *g* for 5 min, and supernatant was carefully removed before resuspending the MEF cell pellet in a warm solution of media containing high-glucose Dulbecco’s modified Eagle medium (DMEM) with l-glutamine (catalog no. 11965-084, GIBCO), supplemented with 10% FBS (catalog no. 16000-044, GIBCO), 100 units/ml penicillin, 100 units/ml streptomycin, and 1 mM sodium pyruvate (catalog no. 11360-070, GIBCO). MEFs were stored at −80°C.

### Cell Culture and Plasmid Transfection

Mouse embryonic fibroblast cells were cultured at 37°C with 5% CO_2_ in high-glucose DMEM with l-glutamine, supplemented with 10% FBS, 100 units/ml penicillin, 100 units/ml streptomycin, and 1 mM sodium pyruvate. Cells were grown to 80–100% confluence before being split using 0.25% Trypsin-EDTA with phenol red (catalog no. 25200-056, GIBCO). MEFs were transfected with plasmid construct(s) using Lipofectamine LTX (catalog no. 15338-100, Invitrogen) and Opti-MEM I Reduced Media Serum (catalog no. 31985-062, Invitrogen) according to the manufacturer’s instructions. Following transfections, cells were collected for RNA isolation and quantitative RT-PCR.

### RNA Isolation and cDNA Synthesis

Approximately, 1 × 10^5^ cells were transfected with 2 μg of the 3111T or 3111C versions of the *CLOCK* SNP. Seventy-two hours following transfection with expression constructs, cells were selected using 100 μg/ml Geneticin Selective Antibiotic (catalog no. 10131-035, GIBCO) to generate stable lines. After selection, RNA from the MEFs was isolated using TRIzol reagent (catalog no. 15596-026, Ambion), according to the manufacturer’s instructions. Briefly, cells were treated with TRIzol reagent and chloroform, and RNA was isolated with the addition of the carrier glycogen, followed by precipitation in isopropanol and centrifugation. Seventy-five percent ethanol was used to wash the RNA pellet, which was re-suspended in nuclease-free H_2_O. Total RNA (200 ng) was treated with DNase I (Catalog no. 18068-015, Invitrogen), and then reverse-transcribed to cDNA using the SuperScript III First-Strand Synthesis System (catalog no. 18080-051, Invitrogen) according to manufacturer’s protocols.

### Quantitative Real-Time PCR

Real-time quantitative polymerase chain reaction (RT-PCR) was completed using the 7900HT Fast Real-Time PCR System (catalog no. 4329003, Applied Biosystems). cDNA samples were prepared with the Power SYBR Green qPCR Master Mix (catalog no. 4367659, Applied Biosystems) along with oligo primers for *hCLOCK* (forward primer: 5′-ATGGGCCAGGTGGTGACTGCAT-3′, reverse primer: 5′-TGACCCAGCCACCGCAACAAT-3′), *G418-resistance gene* present in pcDNA3.1(−) (forward primer: 5′-CGCATGATTGAACAAGATGGATTGC-3′, reverse primer: 5′-GTTCATTCAGGGCACCGGACA-3′), and *mPer2* (forward primer 5′-GAGTGTGTGCAGCGGCTTAG-3′, reverse primer 5′-GTAGGGTGTCATGCGGAAGG-3′). PCRs were run in duplicate, followed by a dissociation reaction, and were subjected to agarose gel electrophoresis to determine specificity of the amplified product. Gene expression was quantified using the ΔCT (CT = cycle threshold) method as previously described (44). Gel electrophoresis of the samples was then performed in order to further confirm the correct size of the amplified PCR products. Efficiencies of qPCR primers used in this study were calculated by means of construction of a standard curve as previously described (45). Samples of experimental MEF cDNA were serially diluted and prepared with the Power SYBR Green qPCR Master Mix (catalog no. 4367659, Applied Biosystems). Dilution series from 1:1 to 1:64 were used for transfected genes (*hCLOCK*, *G418*-resistance gene), while dilution series from 1:1 to 1:8 were used for endogenous genes (*mPer2, Npas2*). qPCRs were run in duplicate and followed by a dissociation reaction. Gene expression was quantified using the ΔCT method and plotted against the logarithm of the dilution factor. The calculated efficiencies for the primer sets used are as follows: *hClock* 88%, *G418* 93%, *mPer2* 110%, and *Npas2* 78%. The observed relatively low expression levels of *Npas2* indicate that *CLOCK* KO MEFs do not express high levels of *Npas2* to compensate for the loss of *CLOCK*. The *hCLOCK* and *G418* primer sets used for determination and comparison of *CLOCK*3111T and *CLOCK*3111C levels have similar efficiencies; therefore, we conclude that these findings do not affect interpretation of the data.

### Circadian Gene Expression Assay

Approximately 1 × 10^4^ cells were plated in 12-well plates, and were doubly transfected with 1 μg each of pcDNA3.1(−) (containing the 3111T, 3111C, or no insert), and a plasmid containing *Npas2* shRNA or a Scr shRNA. A caveat of using *Clock* KO MEFs was that previous research has shown that KO of the *Clock* gene can result in increased *Npas2* expression, possibly as a compensatory mechanism (46). Because, as a CLOCK homolog, NPAS2 also regulates *Per2* gene expression, an *Npas2* shRNA was co-transfected along with either versions of the 3111T/C SNP when measuring *Per2* circadian gene expression in qPCR studies. Seventy-two hours following transfection, MEFs were subjected to serum shock to synchronize individual molecular rhythms as previously described (47). Briefly, cells were serum shocked using DMEM supplemented with 50% horse serum for 30 min to induce expression of circadian genes and synchronize the molecular clock (48). After the serum shock treatment, MEFs were switched to recovery media containing high-glucose DMEM with l-glutamine supplemented with 2% B-27 (catalog no. 17504-044, GIBCO), 350 mg/l sodium bicarbonate, 0.25% penicillin/streptomycin, and 10 mM HEPES (catalog no. 15630-106, GIBCO). Cells were collected every 3 h for the next 27 h (e.g., circadian time CT3, CT6, CT9, CT12, etc.) and processed for RNA isolation/cDNA synthesis. Samples were then used to measure circadian gene expression (as described in Section “Quantitative Real-Time PCR”).

### Statistics

All data are presented as the mean ± SEM. Two-way analysis of variance (ANOVA) and Student’s *t*-tests were performed to determine significant differences between experimental groups and time points. Statistical outliers were identified as ±2× SD of the mean and removed prior to further data analysis.

## Results

### *Npas2* Gene Expression in MEF Cells and Knock-Down

**Figure 1:**
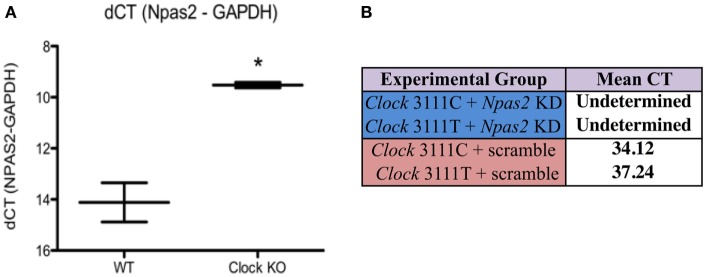
***Npas2* expression in Clock KO and WT MEFs**. **(A)** *Npas2* expression is significantly increased in Clock KO MEFS. *P < 0.05. **(B)** *Npas2* shRNA results in significantly decreased *Npas2* expression as compared with Scramble shRNA (representative data from CT21).

NPAS2, a homolog of CLOCK, can heterodimerize with BMAL1 and positively regulate the transcription of *Per* and *Cry* genes. Since it is possible that *Npas2* may be upregulated in *Clock* KO MEFs, *Npas2* expression in WT and *Clock* KO MEFs was measured using qPCR (Figure 1A). After normalizing CT values to *GAPDH*, we performed a two-tailed unpaired Student’s *t*-test on our preliminary data, which revealed a statistically significant difference between mean ΔCT values [*n* = 2, Figure 1A]. These data show a 4.59-cycle increase in *Npas2* expression in *Clock* KO MEFs compared to WT MEFs (Student’s *t*-test, *P* < 0.05). We used an shRNA to knockdown expression of *Npas2*. *Npas2* shRNA resulted in a decrease in *Npas2* expression as compared with Scr shRNA, as qPCR studies revealed that *Npas2* gene expression was undetected after 40 cycles of PCR in cells expressing the *Npas2* shRNA. Representative data from ZT21 is shown (Figure 1B).

### CLOCK 3111T/C SNP Gene Expression (Single Time Point)

**Figure 2:**
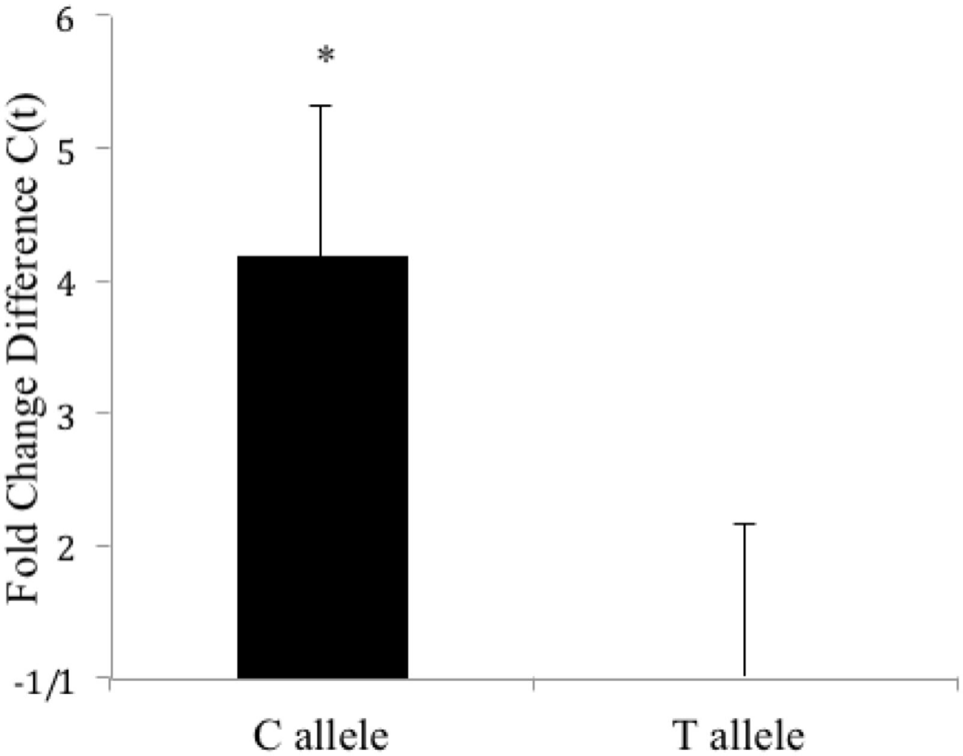
**Fold change difference in *hCLOCK* levels in *Clock*3111T/C-expressing MEFs (single time point)**. *Clock* 3111C gene expression is significantly higher than 3111T expression. *P < 0.05.

To better understand the effects of the *CLOCK* 3111T/C SNP on mRNA expression and stability, we transfected *Clock* KO MEFs with either version of the SNP and sought to measure differences in *CLOCK* mRNA levels between cells expressing the 3111C and 3111T alleles (at a single time point). Using primers for the human *CLOCK* gene, our studies revealed that cells expressing the 3111C allele showed a 4.17-fold increase in *CLOCK* mRNA levels (Student’s *t*-test, *P* < 0.05, *n* = 8, Figure 2). Transfection efficiency was not directly evaluated; we used antibiotic selection to ensure proliferation of only transfected cells. Furthermore, we used the housekeeping gene, *G418* (the gene in the pcDNA plasmid that is responsible for conferring antibiotic resistance to Geneticin) for calculating the dCT.

### Twenty-Four-Hour Gene Expression Levels of *CLOCK* 3111T/C and *Per2*

**Figure 3:**
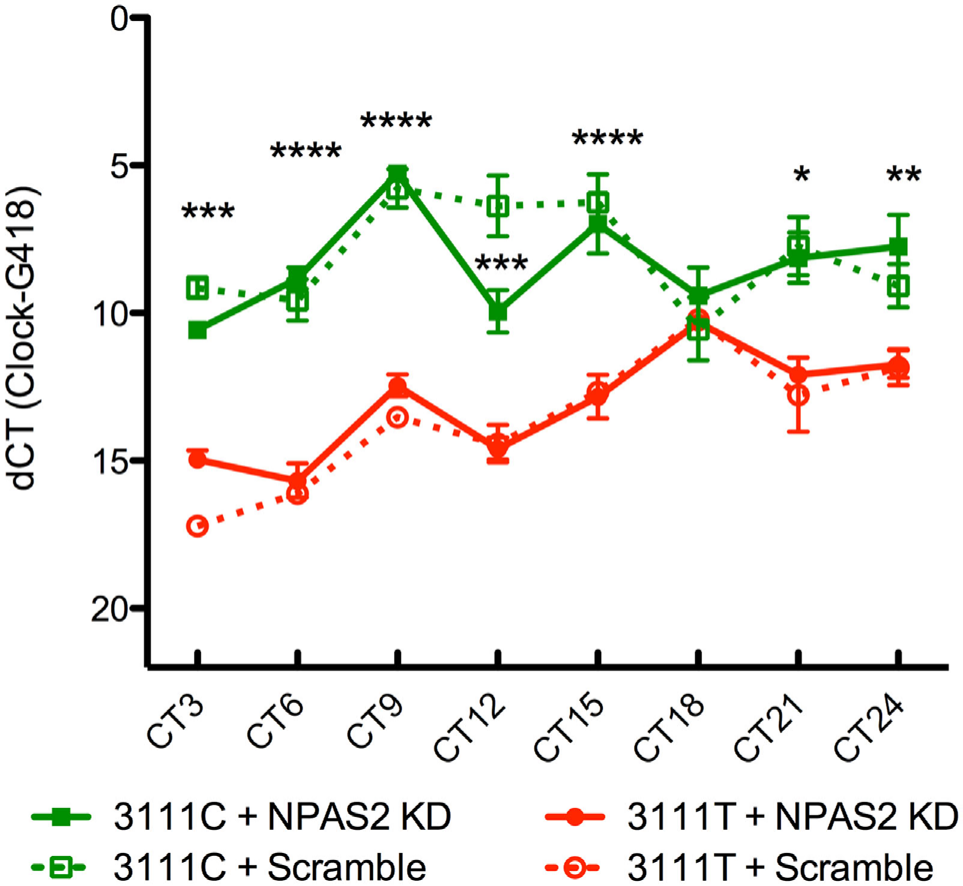
**Twenty-four-hour expression of *hCLOCK* in 3111T/C SNP-expressing MEFs**. Clock 3111C gene expression over 24 h is significantly higher than Clock 3111T expression. ****P < 0.0001, ***P < 0.001, **P < 0.01, *P < 0.05.

To test the functional implications of the *CLOCK* 3111T/C SNP on *CLOCK* mRNA expression and stability over a 24-h time period, we transfected *CLOCK* KO MEFs with either version of the SNP and sought to determine if there were differences in *CLOCK* mRNA levels between cells expressing the 3111C and 3111T alleles. Transfection efficiency was not directly evaluated; we used the housekeeping gene, *G418* (the gene in the pcDNA plasmid that is responsible for conferring antibiotic resistance to geneticin) for calculating the dCT. Our findings show that MEFs transfected with the 3111C construct showed significantly increased levels of *CLOCK* mRNA at every time point measured, excluding CT18 (*n* = 4/group/timepoint, Figure 3). Using a two-way ANOVA, we found a highly significant SNP × time interaction; SNP × time [*F* = (21, 93) = 5.34, *P* < 0.0001]. The main effect of the SNP was highly significant; [*F* = (3, 93) = 156.45, *P* < 0.0001], as was the main effect of time; [*F* = (7, 93) = 16.68, *P* < 0.0001]. Bonferroni *post hoc* test results reveal significant differences in *CLOCK* expression in MEFs co-transfected with 3111T or C SNP constructs (along with *Npas2* shRNA; Figure 3). We were primarily interested in these group comparisons (due to the upregulation of *Npas2* in *Clock* KO MEFs). There were no significant differences in *CLOCK* expression seen in cells transfected with or without the *Npas2* shRNA.

**Figure 4:**
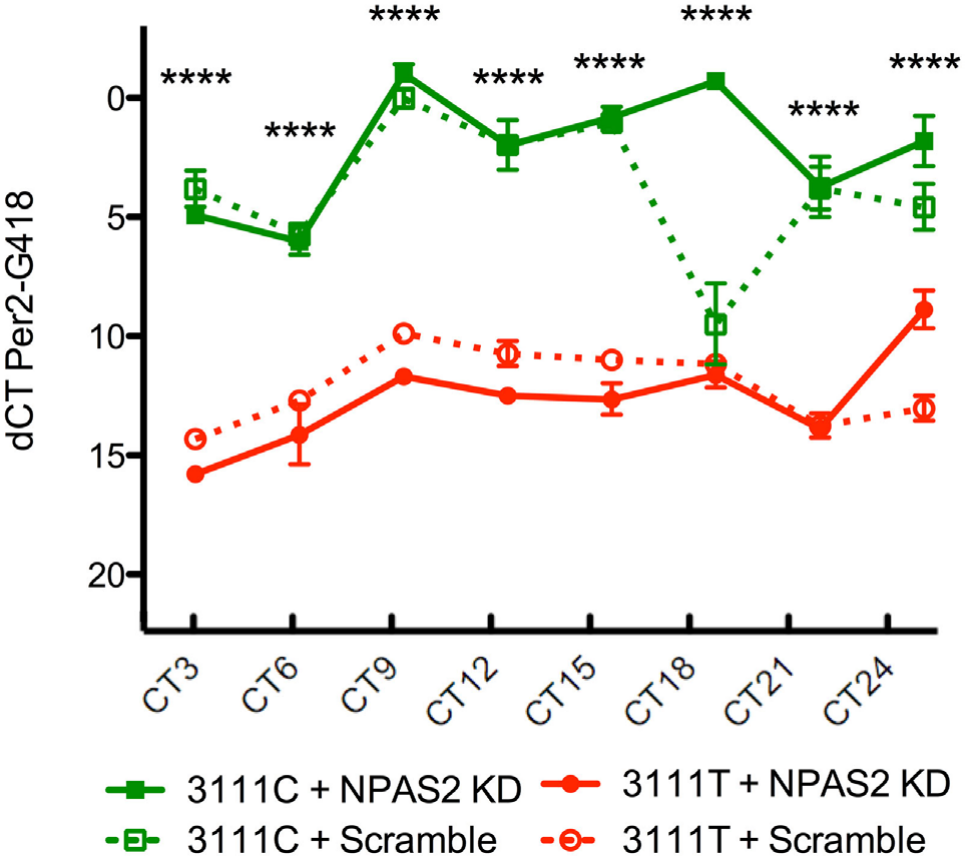
**Twenty-four-hour expression of *Per2* in 3111T/C SNP-expressing MEFs**. *Clock*3111C gene expression over 24 h is significantly higher than *Clock*3111T expression. ****P < 0.0001.

To test the *CLOCK* 3111T/C SNP’s effects on CLOCK protein function, 24-h *Per2* expression was measured in *Clock* KO MEFs co-transfected with the 3111T or 3111C constructs and the *Npas2* shRNA (*n* = 4/group/timepoint, Figure 4). As a downstream transcriptional target of the CLOCK protein, *Per2* is expressed as part of a transcriptional–translational feedback loop in the molecular *clock*. Therefore, alterations to *Per2* expression indirectly reveal differences in CLOCK’s transcriptional activity between cells expressing the T or C variant of the SNP. *Npas2* shRNA plasmid was co-transfected along with the human *CLOCK* constructs in order to eliminate the possible confound of *Per2* expression driven by NPAS2. Using a two-way ANOVA, we found a significant interaction between SNP and time: SNP × time [*F* = (21, 95) = 8.52, *P* < 0.0001]. We also found that the main effect of the SNP was highly significant; [*F* = (3, 95) = 523.66, *P* < 0.0001]. The main effect of time was also significant; [*F* = (7, 95) = 23.67, *P* < 0.0001]. Bonferroni *post hoc* testing revealed that in MEFs expressing the *CLOCK* 3111C variant and *Npas2* shRNA, expression of *Per2* mRNA is significantly increased at every time point measured.

## Discussion

Severe circadian rhythm disruptions are prominent symptoms in mood disorders, including BD and major depression. There is mounting pre-clinical and clinical data, suggesting that circadian genes play a role in a number of disease parameters, such as age of onset (27, 28). Here, we sought to study the implications of the *CLOCK* 3111T/C SNP, a polymorphism located in the 3′-UTR that has been shown to associate with increased frequency and severity of manic episodes and actimetric disturbances in BD patients (2, 31, 32). Alterations to the 3′-UTR have been shown to engage in transcriptional and translational regulation through various pathways, including those involving mRNA stability and degradation (49). In addition, previous animal research has found that reductions in CLOCK expression or function produces increased locomotor and drug- and alcohol-seeking behavior, and decreased depression- and anxiety-like behavior – hallmarks of bipolar mania (1, 36–38). Therefore, we sought to test the hypothesis that the 3111C variant of the 3111T/C SNP would result in reduced *CLOCK* expression and alter *Per2* expression (as a measure of altered CLOCK transcriptional activity) when compared to the 3111T allele. Using cell culture and qPCR, we found that the 3111C variant results in significantly increased *CLOCK* and *Per2* mRNA expression over 24 h.

These findings are very exciting as there is a clear dearth in the literature of molecular research of this SNP. The possibility that a clinically relevant SNP results in increased *CLOCK* expression is important to many interesting genes, including related circadian genes as well as those outside of the molecular clock. For example, in this study, we report that *Per2*, a gene positively regulated by CLOCK, exhibits significantly elevated expression over 24 h in cells expressing the *CLOCK* 3111C SNP. This suggests that CLOCK protein levels are also increased.

Changes in expression of these related circadian genes may have important implications for rhythms of critical physiological processes. Sleep/wake, hormonal, body temperature, and blood pressure rhythms, for instance, are all influenced by molecular clockwork. These changes may also be important in the understanding and development of effective treatments for mood disorders, as current therapies have been shown to strongly phase-shift circadian rhythms. For example, selective serotonin reuptake inhibitors (SSRI’s) and morning-light therapy produce circadian phase shift advances in the SCN, while lithium and valproate induce phase shift delays and period-lengthening (50–54).

Outside of the molecular clock, changes in *CLOCK* expression may have tremendous implications for a wide variety of genes that clearly play an important role in regulating mood and behavior. Tyrosine hydroxylase (TH), dopamine receptor D3 (Drd3), cholecystokinin (CCK), and many other regulators of dopaminergic transmission are under transcriptional control by the CLOCK protein (46, 55, 56). Additionally, mice with an induced mutation of the *Clock* gene, which results in overexpression of a truncated CLOCK protein with dominant-negative function, express a behavioral profile strongly resembling BD patients in the manic state that can be reversed using chronic lithium treatment (1). These mice show increased dopamine synthesis and dopamine firing in the ventral tegmental area (VTA), a region featuring important dopaminergic projections that have been implicated in psychiatric disorders (39). This includes an increase in TH expression in the VTA (36). Most of the manic-like behavioral phenotypes of these mutant mice are rescued with the induced expression of a functional CLOCK protein in the VTA, while knockdown of *Clock* expression in the VTA in wild-type mice produces a “mixed state” of reduced anxiety- but increased depression-like behavior (1, 40, 42). Taking this into consideration, it becomes easy to see how a SNP affecting *Clock* expression can have major functional consequences for a number of diseases.

It is still unclear as to how *CLOCK* mRNA levels are increased in cells expressing the 3111C variant. Changes in mRNA levels for 3111C could be due to increased stability of the mRNA. One possible mechanism to explain the increased mRNA stability is that the 3′-UTR hosts several regulatory elements controlling stability, one of which is binding sites for microRNAs (miRNAs). miRNAs are short, untranslated sequences of RNA that bind specific RNA sequences and, once bound, decrease mRNA transcript stability (57). The 3′-UTR of the *CLOCK* gene also features a binding site for miRNA-182 less than 40 bases from the 3111T/C SNP that may affect stability of the mRNA by preventing or activating degradation mechanisms. It is possible that the 3111C variant may interfere with this site, reducing miRNA-182 binding and thereby increasing stability and elevating levels of *CLOCK* mRNA.

Our studies show that the *CLOCK* 3111T/C SNP has important functional consequences by increasing *CLOCK* and *Per2* mRNA expression over 24 h. These changes may be due to a number of different mechanisms, including miRNA-182 binding sites in the 3′-UTR. In the future, we plan to investigate whether miRNA-182 binding is altered in the 3111T/C SNP. Additional future studies may focus on behavioral studies involving transgenic mice that express either version of the 3111T/C SNP. Given that our studies report altered *Per2* expression, the clinical relevance of the SNP may also be further studied by examining whether carriers of the 3111C allele respond differently to mood stabilizers that are known to induce changes in circadian rhythm phase and period. These results directly help to further characterize the functional implications of this clinically relevant SNP, and because the 3111T/C SNP is only one polymorphism of many identified for the *CLOCK* gene that associates with BD, they provide a stepping off point for future SNP studies in this model.

## Author Contributions

AO, SM, and CM designed the experiments. AO, KP, PP, GK, EF, SM, and HC performed research; KP, AO, GK, EF, and CM analyzed data. AO, KP, GK, and CM wrote the manuscript.

## Conflict of Interest Statement

The authors declare no conflicts of interest associated with this manuscript. This research was conducted in the absence of any commercial or financial relationships that could be construed as a potential conflict of interest.

## References

[1] Roybal K Theobold D Graham A DiNieri JA Russo SJ Krishnan V Mania-like behavior induced by disruption of CLOCK. Proc Natl Acad Sci U S A (2007) 104(15):6406–11. 10.1073/pnas.0609625104 17379666 PMC1851061

[2] Benedetti F Serretti A Colombo C Barbini B Lorenzi C Campori E Influence of CLOCK gene polymorphism on circadian mood fluctuation and illness recurrence in bipolar depression. Am J Med Genet B Neuropsychiatr Genet (2003) 123B:23–6. 10.1002/ajmg.b.20038 14582141

[3] Kupfer DJ Angst J Berk M Dickerson F Frangou S Frank E Advances in bipolar disorder: selected sessions from the 2011 International Conference on Bipolar Disorder. Ann N Y Acad Sci (2011) 1242(1):1–25. 10.1111/j.1749-6632.2011.06336.x 22191553

[4] Kleine-budde K Touil E Moock J Bramesfeld A Kawohl W Rössler W. Cost of illness for bipolar disorder: a systematic review of the economic burden. Bipolar Disord (2014) 16(4):337–53. 10.1111/bdi.12165 24372893

[5] Pini S de Queiroz V Pagnin D Pezawas L Angst J Eur Neuropsychopharmacol (2005) 15(4):425–34. 15935623 10.1016/j.euroneuro.2005.04.011

[6] McClung CA. Circadian genes, rhythms and the biology of mood disorders. Pharmacol Ther (2007) 114(2):222–32. 10.1016/j.pharmthera.2007.02.003 17395264 PMC1925042

[7] McClung CA. How might circadian rhythms control mood? Let me count the ways. Biol Psychiatry (2013) 74(4):242–9. 10.1016/j.biopsych.2013.02.019 23558300 PMC3725187

[8] Post RM. Treatment of bipolar depression: evolving recommendations. Psychiatr Clin North Am (2016) 39(1):11–33. 10.1016/j.psc.2015.09.001 26876316

[9] Szmulewicz A Samamé C Caravotta P Martino DJ Igoa A Hidalgo-Mazzei D Behavioral and emotional adverse events of drugs frequently used in the treatment of bipolar disorders: clinical and theoretical implications. Int J Bipolar Disord (2016) 4(1):6. 10.1186/s40345-016-0047-3 26879750 PMC4754238

[10] Vieta E Hidalgo DH Treatment resistant bipolar depression. Eur Psychiatry (2015) 30(1):58 10.1016/S0924-9338(15)30048-125169444

[11] Atkinson M Kripke DF Wolf SR. Autorhythmometry in manic-depressives. Chronobiologia (1975) 2(4):325–35. 1222678

[12] Dokucu ME Yu L Taghert PH. Lithium- and valproate-induced alterations in circadian locomotor behavior in *Drosophila*. Neuropsychopharmacology (2005) 30:2216–24. 10.1038/sj.npp.1300764 15956996

[13] Gould TD Manji HK. Glycogen synthase kinase-3: a putative molecular target for lithium mimetic drugs. Neuropsychopharmacology (2005) 30(7):1223–37. 15827567 10.1038/sj.npp.1300731

[14] Iwahana E Akiyama M Miyakawa K Uchida A Kasahara J Fukunaga K Effect of lithium on the circadian rhythms of locomotor activity and glycogen synthase kinase-3 protein expression in the mouse suprachiasmatic nuclei. Eur J Neurosci (2004) 19(8):2281–7. 10.1111/j.0953-816X.2004.03322.x 15090054

[15] Johnsson A Engelmann W Pflug B Klemke W. Period lengthening of human circadian rhythms by lithium carbonate, a prophylactic for depressive disorders. Int J Chronobiol (1983) 8(3):129–47. 6862696

[16] Klemfuss H. Rhythms and the pharmacology of lithium. Pharmacol Ther (1992) 56(1):53–78. 10.1016/0163-7258(92)90037-Z 1297145

[17] Kripke DF Mullaney DJ Atkinson M Wolf S. Circadian rhythm disorders in manic-depressives. Biol Psychiatry (1978) 13(3):335–51. 667233

[18] Takahashi JS Shimomura K Kumar V. Searching for genes underlying behavior: lessons from circadian rhythms. Science (2008) 322(5903):909–12. 10.1126/science.1158822 18988844 PMC3744585

[19] Kripke DF Nievergelt CM Joo E Shekhtman T Kelsoe JR. Circadian polymorphisms associated with affective disorders. J Circadian Rhythms (2009) 7:2. 10.1186/1740-3391-7-2 19166596 PMC2661876

[20] Lamont EW Legault-Coutu D Cermakian N Boivin DB. The role of circadian clock genes in mental disorders. Dialogues Clin Neurosci (2007) 9(3):333–42. 17969870 10.31887/DCNS.2007.9.3/elamont PMC3202489

[21] Lamont EW Coutu DL Cermakian N Boivin DB. Circadian rhythms and clock genes in psychotic disorders. Isr J Psychiatry Relat Sci (2010) 47(1):27–35. 20686197

[22] Mansour HA Wood J Logue T Chowdari KV Dayal M Kupfer DJ Association study of eight circadian genes with bipolar I disorder, schizoaffective disorder and schizophrenia. Genes Brain Behav (2006) 5(2):150–7. 10.1111/j.1601-183X.2005.00147.x 16507006

[23] Mansour HA Talkowski ME Wood J Chowdari KV McClain L Prasad K Association study of 21 circadian genes with bipolar I disorder, schizoaffective disorder, and schizophrenia. Bipolar Disord (2009) 11(7):701–10. 10.1111/j.1399-5618.2009.00756.x 19839995 PMC3401899

[24] Sjoholm LK Backlund L Cheteh EH Ek IR Frisen L Schalling M CRY2 is associated with rapid cycling in bipolar disorder patients. PLoS One (2010) 5(9):e12632. 10.1371/journal.pone.0012632 20856823 PMC2939397

[25] Soria V Martinez-Amoros E Escaramis G Valero J Perez-Egea R Garcia C Differential association of circadian genes with mood disorders: CRY1 and NPAS2 are associated with unipolar major depression and CLOCK and VIP with bipolar disorder. Neuropsychopharmacology (2010) 35(6):1279–89. 10.1038/npp.2009.230 20072116 PMC3055337

[26] Harvey AG. Sleep and circadian functioning: critical mechanisms in the mood disorders? Annu Rev Clin Psychol (2011) 7:297–319. 10.1146/annurev-clinpsy-032210-104550 21166537

[27] Benedetti F Dallaspezia S Colombo C Pirovano A Marino E Smeraldi E. A length polymorphism in the circadian clock gene Per3 influences age at onset of bipolar disorder. Neurosci Lett (2008) 445(2):184–7. 10.1016/j.neulet.2008.09.002 18789374

[28] Nievergelt CM Kripke DF Barrett TB Burg E Remick RA Sadovnick AD Suggestive evidence for association of the circadian genes PERIOD3 and ARNTL with bipolar disorder. Am J Med Genet B Neuropsychiatr Genet (2006) 141B(3):234–41. 10.1002/ajmg.b.30252 16528748 PMC2651679

[29] McCarthy MJ Nievergelt CM Kelsoe JR Welsh DK. A survey of genomic studies supports association of circadian clock genes with bipolar disorder spectrum illnesses and lithium response. PLoS One (2012) 7(2):e32091. 10.1371/journal.pone.0032091 22384149 PMC3285204

[30] McGuffin P Rijsdijk F Andrew M Sham P Katz R Cardno A. The heritability of bipolar affective disorder and the genetic relationship to unipolar depression. Arch Gen Psychiatry (2003) 60(5):497–502. 10.1001/archpsyc.60.5.497 12742871

[31] Benedetti F Dallaspezia S Fulgosi MC Lorenzi C Serretti A Barbini B Actimetric evidence that CLOCK 3111 T/C SNP influences sleep and activity patterns in patients affected by bipolar depression. Am J Med Genet B Neuropsychiatr Genet (2007) 144B(5):631–5. 10.1002/ajmg.b.30475 17221848

[32] Serretti A Cusin C Benedetti F Mandelli L Pirovano A Zanardi R Insomnia improvement during antidepressant treatment and CLOCK gene polymorphism. Am J Med Genet B Neuropsychiatr Genet (2005) 137B(1):36–9. 10.1002/ajmg.b.30130 15952199

[33] Desan PH Oren DA Malison R Price LH Rosenbaum J Smoller J Genetic polymorphism at the CLOCK gene locus and major depression. Am J Med Genet (2000) 96(3):418–21. 10.1002/1096-8628(20000612)96:3<418::AID-AJMG34>3.0.CO;2-S 10898925

[34] Hentze MW Kuhn LC. Molecular control of vertebrate iron metabolism: mRNA-based regulatory circuits operated by iron, nitric oxide, and oxidative stress. Proc Natl Acad Sci U S A (1996) 93:8175–82. 10.1073/pnas.93.16.8175 8710843 PMC38642

[35] Zaidi SH Malter JS Amyloid precursor protein mRNA stability is controlled by a 29-base element in the 3c-untranslated region. J Biol Chem (1994) 269:24007–13. 7929051

[36] McClung CA Sidiropoulou K Vitaterna M Takahashi JS White FJ Cooper DC Regulation of dopaminergic transmission and cocaine reward by the Clock gene. Proc Natl Acad Sci U S A (2005) 102(26):9377–81. 10.1073/pnas.0503584102 15967985 PMC1166621

[37] Ozburn AR Larson EB Self DW McClung CA. Cocaine self-administration behaviors in Clock▵19 mice. Psychopharmacology (Berl) (2012) 223(2):169–77. 10.1007/s00213-012-2704-2 22535308 PMC3670183

[38] Ozburn AR Falcon E Mukherjee S Gillman A Arey R Spencer S The role of clock in ethanol-related behaviors. Neuropsychopharmacology (2013) 38(12):2393–400. 10.1038/npp.2013.138 23722243 PMC3799058

[39] Coque L Mukherjee S Cao JL Spencer S Marvin M Falcon E Specific role of VTA dopamine neuronal firing rates and morphology in the reversal of anxiety-related, but not depression-related behavior in the Clock▵19 mouse model of mania. Neuropsychopharmacology (2011) 36(7):1478–88. 10.1038/npp.2011.33 21430648 PMC3096816

[40] Dzirasa K Coque L Sidor MM Kumar S Dancy EA Takahashi JS Lithium ameliorates nucleus accumbens phase-signaling dysfunction in a genetic mouse model of mania. J Neurosci (2010) 30(48):16314–23. 10.1523/JNEUROSCI.4289-10.2010 21123577 PMC3165036

[41] Ozburn AR Falcon E Twaddle A Nugent AL Gillman AG Spencer SM Direct regulation of diurnal Drd3 expression and cocaine reward by NPAS2. Biol Psychiatry (2015) 77(5):425–33. 10.1016/j.biopsych.2014.07.030 25444159 PMC4315729

[42] Mukherjee S Coque L Cao JL Kumar J Chakravarty S Asaithamby A Knockdown of Clock in the ventral tegmental area through RNA interference results in a mixed state of mania and depression-like behavior. Biol Psychiatry (2010) 68(6):503–11. 10.1016/j.biopsych.2010.04.031 20591414 PMC2929276

[43] Jozefczuk J Drews K Adjaye J. Preparation of mouse embryonic fibroblast cells suitable for culturing human embryonic and induced pluripotent stem cells. J Vis Exp (2012) (64). 10.3791/3854 22760161 PMC3471299

[44] Tsankova NM Kumar A Nestler EJ. Histone modifications at gene promoter regions in rat hippocampus after acute and chronic electroconvulsive seizures. J Neurosci (2004) 24(24):5603–10. 10.1523/JNEUROSCI.0589-04.2004 15201333 PMC6729334

[45] Bustin SA Benes V Garson JA Hellemans J Huggett J Kubista M The MIQE guidelines: minimum information for publication of quantitative real-time PCR experiments. Clin Chem (2009) 55(4):611–22. 10.1373/clinchem.2008.112797 19246619

[46] Ozburn AR Falcon E Twaddle A Nugent AL Gillman AG Spencer SM Direct regulation of diurnal Drd3 expression and cocaine reward by NPAS2. Biol Psychiatry (2015) 77(5):425–33. 10.1016/j.biopsych.2014.07.030 25444159 PMC4315729

[47] Izumo M Johnson CH Yamazaki S. Circadian gene expression in mammalian fibroblasts revealed by real-time luminescence reporting: temperature compensation and damping. Proc Natl Acad Sci U S A (2003) 100(26):16089–94. 10.1073/pnas.2536313100 14657355 PMC307697

[48] Balsalobre A Damiola F Schibler U. A serum shock induces circadian gene expression in mammalian tissue culture cells. Cell (1998) 93(6):929–37. 10.1016/S0092-8674(00)81199-X 9635423

[49] Mignone F Gissi C Liuni S Pesole G. Untranslated regions of mRNAs. Genome Biol (2002) 3(3):REVIEWS0004. 10.1186/gb-2002-3-3-reviews0004 11897027 PMC139023

[50] Dudley TE Dinardo LA Glass JD. *In vivo* assessment of the midbrain raphe nuclear regulation of serotonin release in the hamster suprachiasmatic nucleus. J Neurophysiol (1999) 81(4):1469–77. 10200183 10.1152/jn.1999.81.4.1469

[51] Lewy AJ Lefler BJ Emens JS Bauer VK The circadian basis of winter depression. Proc Natl Acad Sci U S A (2006) 103(19):7414–9. 10.1073/pnas.0602425103 16648247 PMC1450113

[52] Lewy AJ Rough JN Songer JB Mishra N Yuhas K Emens JS. The phase shift hypothesis for the circadian component of winter depression. Dialogues Clin Neurosci (2007) 9(3):291–300. 17969866 10.31887/DCNS.2007.9.3/alewy PMC3202495

[53] Sprouse J Braselton J Reynolds L. Fluoxetine modulates the circadian biological clock via phase advances of suprachiasmatic nucleus neuronal firing. Biol Psychiatry (2006) 60(8):896–9. 10.1016/j.biopsych.2006.03.003 16631132

[54] Terman M Jiuan Su T Circadian rhythm phase advance with dawn simulation treatment for winter depression. J Biol Rhythms (2010) 25(4):297–301. 10.1177/0748730410374000 20679499

[55] Arey RN Enwright JF III Spencer SM Falcon E Ozburn AR Ghose S An important role for cholecystokinin, a CLOCK target gene, in the development and treatment of manic-like behaviors. Mol Psychiatry (2014) 19(3):400. 10.1038/mp.2013.12 23399917 PMC3783638

[56] Sidor MM Spencer SM Dzirasa K Parekh PK Tye KM Warden MR Daytime spikes in dopaminergic activity drive rapid mood-cycling in mice. Mol Psychiatry (2015) 20(11):1479–80. 10.1038/mp.2015.8 25687774 PMC5152674

[57] Kuersten S Goodwin EB. The power of the 3′ UTR: translational control and development. Nat Rev Genet (2003) 4(8):626–37. 10.1038/nrg1125 12897774

